# A Comparative Analysis of Clinical Presentation, Prognosis and Outcomes in Paralytic Dogs with a Compressive and a Contusive Intervertebral Disc Disease

**DOI:** 10.3390/vetsci12030287

**Published:** 2025-03-19

**Authors:** Anna Kurtscheidt, Stefan Rupp, Ute Müller, Franck Forterre

**Affiliations:** 1 Division of Small Animal Surgery, Department of Clinical Veterinary Medicine, Vetsuisse Faculty, University of Bern, 3012 Bern, Switzerland; 2 Neurology Department, Tierärzte IVC Evidensia GmbH Tierklinik Hofheim, 65719 Hofheim am Taunus, Germany; 3 Institute of Animal Science, Department Physiology, University of Bonn, 53113 Bonn, Germany

**Keywords:** ANNPE, canine IVDE, decompressive surgery, IVDD, compression, contusion

## Abstract

This retrospective study aims to investigate whether dogs with a contusive and dogs with a compressive intervertebral disc disease share comparable outcomes and prognoses when presenting with the same neurological grade. A total of 50 dogs with IVDE (compression group) and 45 dogs with ANNPE (contusion group) were included in this study. The protocol involved a thorough review of the medical records and CT/MRI images of the included dogs. Data encompassing signalment, initial clinical signs, post-diagnosis treatment and short-term outcomes (less than 8 months) were collected from the patient records. Information on long-term outcomes (more than 8 months) was obtained through interviews with the owners. Dogs in the compression group displayed clinical signs at a statistically significant younger age and were more likely to experience deteriorating neurological deficits compared to the contusion group. Conversely, dogs with contusions had a significantly higher incidence of external trauma history and vocalization at the onset of clinical signs. In both groups, the most affected spinal cord segment was T3-L3. The overall recovery rate to regain independent ambulation was 71% in the contusion group vs. 80% in the compression group. While some of the examined parameters revealed differences, most did not significantly differ between the groups.

## Simple Summary

Intervertebral disc disease is a common cause of spinal dysfunction in dogs, leading to pain, motor impairment and proprioceptive deficits. Two major forms, acute compressive intervertebral disc extrusion and acute non-compressive nucleus pulposus extrusion, present with similar clinical signs but differ in pathophysiology. Despite extensive research, the exact etiology of both conditions remains unclear. This retrospective study compares the clinical presentation, outcome and prognosis of dogs with compressive intervertebral disc disease (IVDE) and contusive intervertebral disc disease (ANNPE). The study hypothesis is that in dogs presenting with the same neurological grade, there would be no significant difference in their ability to regain ambulation when matched by neurological grade.

## 1. Introduction

Intervertebral disc disease (IVDD) constitutes a frequent source of spinal cord dysfunction seen in dogs [1], potentially leading to acute or chronic neuropathic pain, motor impairment and even profound proprioceptive deficits [2]. Acute non-compressive nucleus pulposus extrusion (ANNPE) and acute compressive intervertebral disc extrusion (IVDE) are two types of thoracolumbar disc disease, both provoking analogous clinical manifestations such as limb weakness or paralysis [3,4]. While IVDE is hallmarked by spinal cord compression resulting from disc material [5], ANNPE denotes a spinal cord contusion suspected to be induced by high kinetic energy impact [3].

Canine intervertebral disc disease has been the subject of extensive research spanning decades. Nonetheless, the complexity of IVDD is not yet fully understood due to diverse vertebral and intervertebral disc features, and the precise etiology of IVDE and ANNPE remains elusive [5,6].

It is known that several predispositions for intervertebral disc degeneration exist, encompassing specific breeds, age and genetic attributes. In 1967, Priester described a susceptibility among diverse chondrodystrophic breeds, such as the French Bulldog, Dachshund and Pekingese [7]. Subsequent studies have consistently corroborated these findings [6,8,9].

The contemporary management of dogs with compressive IVDD encompasses either a conservative or surgical approach. Non-surgical treatment includes cage rest, analgesia or anti-inflammatory drugs and intense physiotherapy [1,3,5,10,11,12,13,14,15,16,17,18,19,20,21,22,23,24,25]. Times for recovery after ANNPE varies significantly, likely influenced by the severity of the spinal cord injury [22]. The reported median hospitalization durations range from 3.0 [24] to 4.5 [22] days, with time to independent ambulation ranging from 2.0 [24] to 16.5 [22] days. Maximum clinical improvement may take several months, with a median time of 2 months reported in one study [24].

While some studies have compared two different contusive spinal cord diseases [24,26], and others have examined conservative versus surgical management in compressive IVDD, no investigation has yet been published that directly compares a compressive condition (IVDE) with a contusive condition (ANNPE). However, this classification has its limitations, as neither condition can be classified as purely compressive or contusive [27]. However, based on the existing knowledge of IVDD and ANNPE, it is reasonable to assume that IVDD is predominantly compressive, while ANNPE is primarily contusive. This rationale formed the basis of our classification decision. This retrospective study aims to compare the clinical signs, outcomes and prognoses of plegic dogs attributed to either a mainly compressive (IVDE) or mainly contusive (ANNPE) intervertebral disc disease. We hypothesized, based on previous research, that no statistically significant difference would be found when comparing these variables in dogs with the same neurological grade, specifically in regard to regaining the ability to walk.

## 2. Materials and Methods

### 2.1. Patients

The medical records of dogs presented at the reporting institution between 2017–2023, diagnosed with IVDE or presumptively diagnosed with ANNPE, were subjected to a retrospective evaluation. For initial inclusion, all dogs had to exhibit an acute (<12 h) onset of neurological signs. All neuroanatomical localizations were included. Moreover, the IVDE patients had to undergo surgical treatment after diagnosis. Complete digital records were mandatory for all cases. A total of 429 cases of ANNPE and 1094 cases of IVDE fulfilled the initial criteria. For final inclusion, the patients had to be presented with acute paralysis (Sharp and Wheeler grade 4 or 5), had undergone additional diagnostic imaging (CT or MRI) and had provided owner consent for a treatment trial post-diagnosis. Patients meeting these inclusion criteria were then categorized into two groups.

The first group comprised dogs diagnosed with a compressive intervertebral disc disease (IVDE) through MRI or CT scans and who were treated surgically, while the second group comprised dogs presumptively diagnosed with contusive intervertebral disc disease (ANNPE) based on MRI evaluation.

The exclusion criteria encompassed dogs who were euthanized immediately after diagnosis due to the owner’s decision, those without further diagnostic imaging or those with other spinal cord disorders (e.g., hydrated nucleus pulposus extrusion, fibrocartilaginous embolic myelopathy or neoplasia).

An individual follow-up was conducted for each patient through owner contact, adhering to local data privacy regulations, except for those known to have already been euthanized. For deceased dogs, the cause of death and their last known neurological status were noted. The follow-up time was individual for each patient, depending on the date of diagnosis (with a minimum of at least 8 months to ensure patients with a slower recovery were able to reach a plateau state). Owners of the surviving dogs were asked the same set of questions in order to gather follow-up information, including the current neurological status, timeframe to independent ambulation and follow-up treatment. Upon reviewing all patient histories, 45 dogs were included in the contusive group, while for comparison, a representative severity-of-clinical-signs-matched group comprising 50 dogs with compressive IVDD out of a database containing 583 cases was randomly selected using Microsoft Excel 365.

The data extracted from medical records included the breed, sex, age (current and at presentation), body weight, the time frame from the onset of clinical signs to presentation, neurological examination findings (at presentation and upon discharge) and diagnostic results. Additionally, in the compression group, specific surgery details were noted (e.g., hemilaminectomy, mini-hemilaminectomy, fenestration). The neurological grading employed the Sharp and Wheeler scoring system [1], as outlined in Table 1. The grading system was slightly adapted, as the original one was developed for thoracolumbar disc disease. Information regarding short-term outcomes was obtained from the medical records, including the duration of hospitalization, duration to independent ambulation (ability to walk >10 steps without assistance) and follow-up treatment (medication and physical intervention). Long-term outcome was determined successful by improvement to independent ambulation without fecal or urinary incontinence after a period of 8 months. Conversely, long-term outcomes had been determined unsuccessful if the patient failed to regain conscious walking capability, had persistent fecal or urinary incontinence or was euthanized due to a lack of improvement at the time of the telephone interview.

### 2.2. Diagnostic Imaging (CT and MRI)

All patients underwent uniform general anesthesia protocols. An intravenous catheter was placed in an accessible peripheral vein. The patients were then induced with diazepam (Ziapam, 5 mg/mL, Eucophar, Fontenay-sous-Bois, France) at a dose of 0.5 mg/kg intravenously (IV) alongside with propofol (Narcofol, 10 mg/mL, cp-pharma, Burgdorf, Germany) IV titrated to effect. An endotracheal tube was inserted, and the anesthesia was maintained with a volatile anesthetic (Isofluran CP, 1 ml/mL, cp-pharma, Burgdorf, Germany) at a 1–2.5 Vol% concentration in oxygen. Patients were positioned in standardized dorsal recumbency, adjusting the front limb positioning according to the suspected neuroanatomical localization, and the hind limbs were extended caudally.

Scans were conducted based on neuroanatomical localization: from head to T2, from T2 to L3 or from L3 to S3. The CT scans utilized a 16-slice helical CT scanner (Aquilion^®^, Canon medical systems GmbH, Neuss, Germany), while the MRI employed a 1.5 Tesla unit (Toshiba Vantage ELAN, Toshiba medical systems GmbH, Berlin, Germany). Standard MRI sequences included STIR DOR, T2 sagittal and transversal, alongside T2* sequences. The CT images were standardized with bone and soft tissue windows, and an IV contrast medium (Accupaque, 350 mg/mL, GE Healthcare Handels GmbH, Wien, Austria) was additionally used for evaluation.

### 2.3. Image Analysis

**Figure 1:**
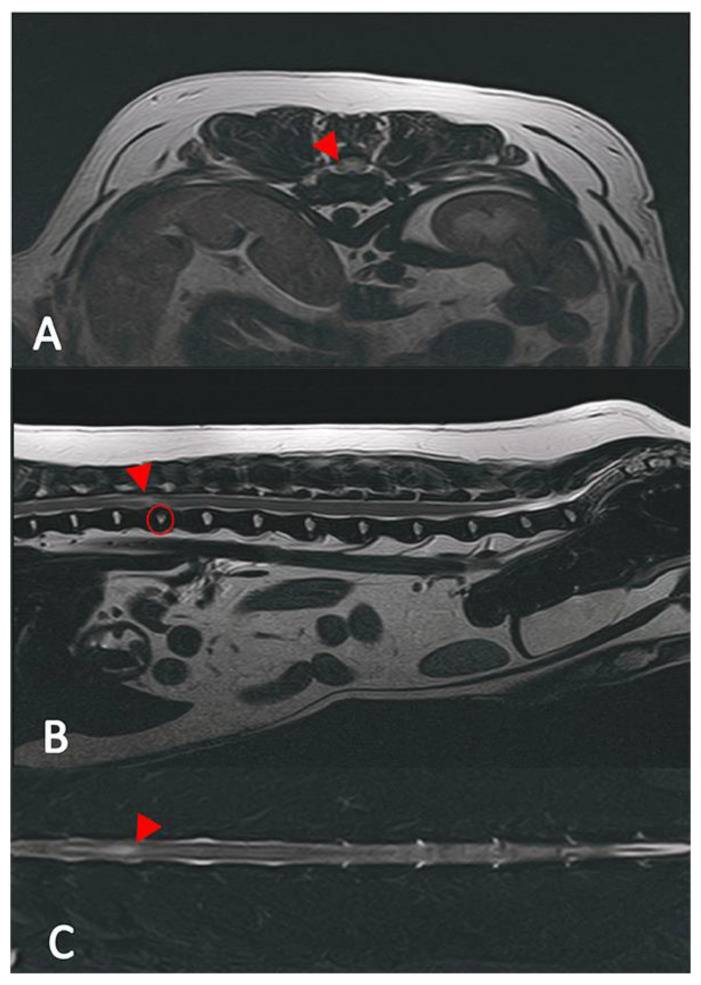
MRI of a dog with presumptive diagnosed ANNPE at the level of T12/13. (**A**) Sagittal, T2-weighted, TSE image with an intramedullary, focal hyperintensity connected to the T12 intervertebral disc space (red arrowhead). (**B**) Transverse, T2-weighted, FSE, with evidence of a slightly degenerated disc (red circle) and a focal hyperintensity associated with the affected intervertebral disc (red arrowhead). (**C**) Dorsal, T2-weighted image, showing the expansion of the intramedullary hyperintensity (red arrowhead).

**Figure 2:**
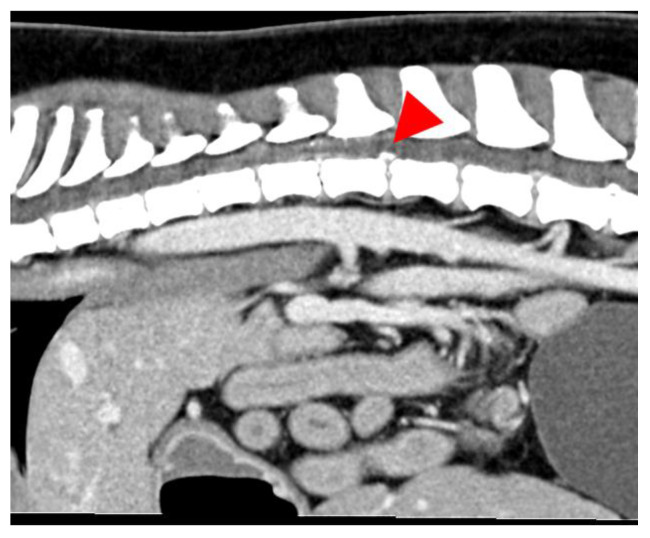
Image of a dog showing calcified disc material inside the vertebral canal. The sagittal plane, soft tissue window and post-contrast CT image with a bone opaque mass are suggestive of extruded disc material at the level of L1–L2 (red arrowhead).

**Figure 3:**
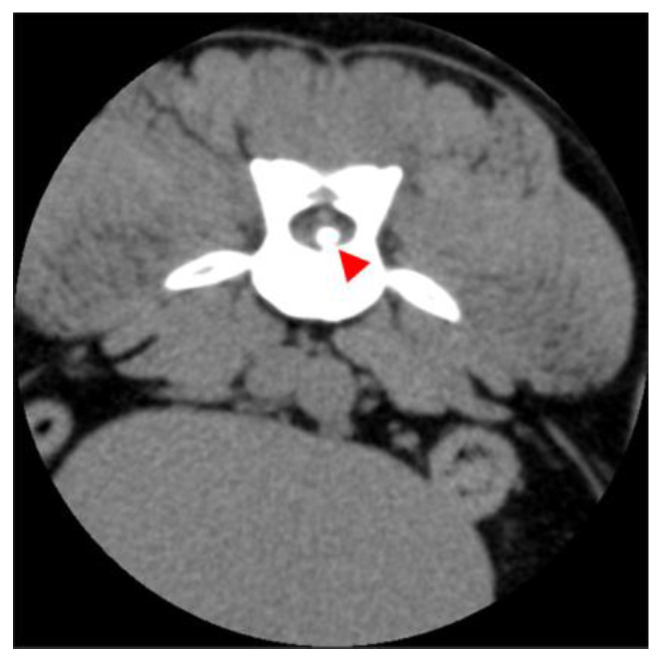
Image of a dog showing calcified disc material inside the vertebral canal. The transverse plane, soft tissue window and post-contrast CT image show a hyperattenuating mass into the vertebral canal at the level of L1–L2 (red arrowhead).

All images were assessed by board-certified veterinary radiologists (ECVDI) using OsiriX MD (OsiriX MD, Version 13.0.1, Pixmeo SARL^©^, Geneva, Switzerland). The MR images were evaluated for intervertebral disc space narrowing, parenchymal changes within the spinal cord associated with a degenerated disc, the presence of disc material in the vertebral canal causing spinal cord compression and the texture of the nucleus pulposus on the T2 images. In the MRI, a reduced volume of the nucleus pulposus coupled with non-compressive extradural material associated with the affected intervertebral disc, the presence of an intramedullary T2 hyperintense lesion (the intensity defined as compared to normal spinal cord parenchyma), whether the lesion was overlying an intervertebral disc space [28], as well as the length of the lesion, were calculated in relation to C6 or L2 vertebral body length [22], which led to a presumptive ANNPE diagnosis Figure 1. A lesion length to vertebral body length of <0.60 was graded as mild, 0.61–0.63 was graded as moderate and >0.63 was graded as severe. Classical signs of intervertebral disc extrusion in the CT scans encompass mineralized material within the spinal canal and protruding soft tissue disc material (Figure 2 and Figure 3) [29,30]. In the case of intervertebral disc extrusion, the severity of the lesion was categorized based on the percentage of reduction in spinal cord diameter, as described by Da Costa et al. [31]. A reduction of <25% was graded as mild, a 25–50% reduction was moderate and a >50% reduction of the spinal cord diameter was graded as severe [31]. In inconclusive cases, additional MRI scans were necessary alongside the initial CT.

### 2.4. Statistical Analysis

Statistical analyses were performed using Microsoft Excel 365 (Microsoft Corporation Version 2303, Redmond, WA, USA, 2023) and SPSS 29 (IBM, Armonk, NY, USA). The descriptive statistics involved comparing the signalment, clinical variables and outcomes between the contusion and compression groups. Prior to generating the descriptive statistics, the normal distribution of each continuous variable was assessed using the Shapiro–Wilk test. For normally distributed continuous variables, the mean ± SD was reported; for the non-normally distributed and ordinal variables, the median was used. Categorical variables were reported as integers and percentages. The independent two-tailed *t*-test was employed to compare continuous variables between the two groups, considering variance homogeneity using Levene’s test. The Pearson’s Chi-squared test was used for categorical data. A *p*-value of ≤0.05 was considered significant.

## 3. Results

### 3.1. Patients

The contusion group consisted of 17 sexually intact females, 9 neutered females, 7 neutered males and 12 sexually intact males with a mean body weight of 23.6 kg ± 10.7 kg and a mean age of 7 years. The represented breeds included Mixed Breed [n = 11 (24%)], Border Collie [n = 4 (9%)], American Staffordshire Terrier [n = 3 (7%)], Australian Shepherd [n = 3 (7%)], French Bulldog [n = 3 (7%)], Labrador Retriever [n = 3 (7%)] and 18 other breeds, with each n = 1 (2%). The compression group encompassed 14 sexually intact females, 15 neutered females, 15 neutered males and 6 sexually intact males with a mean body weight of 11.5 kg ± 10.7 kg and a mean age of 5.5 years. The most frequently presented breeds were Mixed Breed [n = 13 (26%)], French Bulldog [n = 12 (24%)], Dachshund [n = 10 (20%)] and nine other breeds with each n = 1 (2%). The signalment and clinical presentation comparisons are summarized in Table 2 There were no significant differences in sex distribution and mean age between the two groups.

### 3.2. Clinical Signs and Diagnostic Findings

In both cohorts, the dogs presented with a non-ambulatory status, accompanied by or without nociception (contusion group S/W grade 4: 33/45 [73%]; S/W grade 5: 12/45 [27%]; compression group S/W grade 4: 31/50 [62%]; S/W grade 5: 19/50 [38%]). The majority of patients were paraplegic 78/95 (82%), while 16/95 (17%) had monoplegia. Only one dog in the contusion group was tetraplegic due to a lesion at the level of C3/4.

The analysis revealed no statistically significant disparities in the severity of neurological deficits between the groups or within the groups themselves. Dogs with a presumptive ANNPE diagnosis werepresented earlier for veterinary care in comparison to the dogs with IVDE [ANNPE 100% in <12 h after onset of clinical signs (*p* < 0.001)]. Among the 95 dogs, 36 (38%) showed vocalization upon onset of clinical symptoms, and 25 dogs (26%) had a history of external trauma, yet in the majority of cases, no trigger could be observed. Eighteen (72%) of those dogs displayed a combination of trauma and vocalization, all of whom were presumptively diagnosed with ANNPE. Deterioration of neurological symptoms in the first 24 h after onset of the clinical signs was seen 2.6 times more often in the compression group compared to the contusion group.

The most affected neuroanatomical localization was T3-L3, with no significant difference between the two groups (contusion 38/45 84%; compression 44/50 88%), as outlined in Table 2, nor regarding the Sharp and Wheeler grade, as outlined in Table 3. The other neuroanatomical localizations were C1-C5 with one dog in each group (2%), L4-S3 with 6/45 in the contusion group (13%) and 5/50 dogs in the compression group (10%). In this study, it is important to note that there was a disparity observed in the diagnostic imaging findings (*p* < 0.001), which revealed less severe spinal lesions in the dogs presumptively diagnosed with ANNPE compared to those with IVDE but no statistical relationship was found between the severity of the diagnostic imaging findings and clinical signs in both groups (Table 2). Consistent with previous reports, the diagnostic imaging and clinical signs were predominantly lateralized [3,27].

### 3.3. Short-Term and Long-Term Outcomes

A total of 17 out of the 95 dogs included were euthanized. Of these, eight dogs (8.4%) were euthanized at the owner’s request, while three dogs (3%) were euthanized due to a lack of improvement after 14 days based on the owner`s decision after thorough communication with the responsible veterinarian, including one dog with ANNPE and three dogs with IVDE. Additionally, six dogs (6%) were euthanized due to suspected myelomalacia, with three dogs in the contusion and three dogs in the compression groups affected by this condition. The suspected diagnosis of myelomalacia was made due to worsening clinical symptoms and/or repeated MRI scans; no pathological examination was performed due to the owner’s denial. These 17 dogs, comprising 10 with presumptive ANNPE and seven with IVDE, were excluded from all outcome analyses. Additionally, at the time of the interview, eight dogs that were initially included in this study had been euthanized due to either age-related co-morbidities or acute fatal conditions (four in the contusion group and four in the compression group).

Information concerning outcomes was available for 95 included patients. Data were sourced from patient histories and owner contacts. Two cases were not excluded despite unavailable owners due to changes in their contact information, as the required data were accessible. Successful long-term outcomes did not differ between dogs with contusive or compressive IVDD (*p* = 0.79).

The duration of hospitalization was greater in dogs with IVDE compared to those with ANNPE, with a mean of 3.7 days compared to 2.5 days (*p* < 0.001). The incidence of ambulation at discharge did not differ between the two groups: 5/45 (11%) in the contusion group compared to 12/50 (24%) in the compression group (*p* = 0.10), nor did the incidence of independent ambulation within 14 days post-initial diagnosis (*p* = 0.32) as 13/45 (39%) of the dogs presumptively diagnosed with ANNPE regained walking ability 14 days after initial clinical signs (13/33 S/W grade 4 and 0/12 S/W grade 5), whereas 24/50 (48%) of the dogs diagnosed with IVDE were able to walk independently within 14 days (17/31 S/W grade 4 and 7/19 S/W grade 5).

The long-term outcome was successful for 32/45 dogs with presumptive ANNPE (71%) and 40/50 dogs with IVDE (80%) and was considered unsuccessful for three dogs in each group. Among the 32 dogs with successful long-term outcomes, 21 showed persistent mild proprioceptive deficits [17 with ANNPE (38%) and four with IVDE (8%)]. The six patients with unsuccessful long-term outcomes did not regain walking ability [three dogs with ANNPE (7%) and three dogs with IVDE (6%)] or experienced persistent fecal or urinary incontinence [three with ANNPE (7%) and two with IVDE (4%)]. The incidence of persistent urinary or fecal incontinence did not significantly differ between dogs with contusive or compressive IVDD, but the presence of persistent incontinence overlapped with the non-recovery of ambulation except in one IVDE patient, who was continent despite paralysis. Nor did the incidence of regaining independent ambulation differ significantly between both analyzed groups.

## 4. Discussion

This study evaluated 45 dogs with mainly contusive IVDD and 50 dogs with mainly compressive IVDD, with no significant differences in the sex distribution, age or severity of neurological deficits between the groups. The T3-L3 spinal region was the most commonly affected area in both cohorts. ANNPE patients presented for veterinary care sooner and exhibited milder findings upon MRI compared to the compression group that underwent the same diagnostic modality.

The long-term outcomes revealed no significant differences between the groups in terms of regaining ambulation or the occurrence of persistent urinary or fecal incontinence. Although ANNPE dogs had a shorter duration of hospitalization, the overall recovery rates and incidence of persistent neurological deficits were comparable between the two groups. Our data show that dogs with ANNPE were older and had a higher body weight compared to IVDE dogs (Table 2). The previously described breed predispositions were reaffirmed in the compression group, along with the identification of predilection sites for thoracolumbar disc disease [2,5,7,32]. This breed predisposition likely contributed to the significantly lower body weight observed in the compression group.

In diagnostic imaging, the IVDE patients exhibited more pronounced changes compared to the ANNPE patients, which was inconsistent with the clinical signs. With respect to the outcome, no statistically significant difference was observed between the two groups. To find a possible explanation for this outcome, it is crucial to delve into the pathophysiology of these conditions and their treatment options: both ANNPE and IVDE cause significant secondary changes in the spinal cords’ parenchyma. These include hemorrhage, edema, swelling and signs of malacia of white and gray matter [33], which occur due to different primary mechanisms—either high kinetic trauma in ANNPE or predominantly compressive alongside contusive forces from extruded, degenerated disc material in IVDE. Despite these different initial mechanisms, the contusive secondary changes, at least on the clinical level, are similar in nature. The secondary changes trigger similar physiological responses in the spinal cord, such as inflammation, oxidative stress and disruption of the blood–spinal cord barrier. Whether these changes are caused by the direct external trauma of ANNPE or by compression from IVDE, the response and repair mechanisms are analogous.

The age distribution at onset for dogs in both the contusion and compression groups aligned with previous reports [3,22,25,26]. This could be attributed to the distinct pathogenesis of these conditions. IVDE is known to be associated with various pathological changes in affected discs, often linked to breed predisposition or genetic factors. A prime example is the occurrence of premature intervertebral disc degeneration in chondrodystrophic dog breeds, which is linked, for example, with a high allele frequency of the CFA12 FGF4 retrogene [34]. Studies have consistently shown that affected breeds experience disc degeneration at a significantly younger age compared to non-chondrodystrophic breeds [8,9,35,36].

Furthermore, age-related changes in the microstructure and biomechanics of the annulus fibrosus have been reported in various species, including dogs [2,5,27,37]. These changes affect collagen fiber cross-linking and result in decreased water and proteoglycan levels, altering fiber cohesiveness. These degenerative alterations may predispose individuals to annular fiber separation and the formation of annular clefts, making them susceptible to nucleus pulposus extrusion when subjected to mechanical stress, such as trauma or exercise [3,37].

Conversely to the compression group, in the contusion group, there was no confirmation of the previously reported breed predisposition for Greyhounds. However, Border Collies were overrepresented, supporting prior findings [24]. The exact cause of this predisposition remains elusive, but both Greyhounds and Border Collies are highly active and athletic breeds. This correlates with the fact that ANNPE patients predominantly have a history of trauma or strenuous exercise prior to the onset of clinical signs. The distribution of breeds in this study could also reflect breed popularity in Germany, as Border Collies have a substantial presence compared to Greyhounds, according to the “VDH Welpenstatistik 2022” [38]. Although Mixed Breeds were the most frequently represented patients in both groups, this is probably based on the fact that Mixed Breeds generally constituted the largest proportion of the population of presented patients during the observed period (breed statistics from Tierklinik Hofheim 2017–2023).

Compared to the IVDE cases, the ANNPE patients were generally older and presented with higher body weights. This difference in distribution could be because the compression group was dominated by chondrodystrophic breeds, whereas the contusion group was dominated by a mixed population. They also had a higher likelihood of having a history of external trauma, often accompanied by vocalization at the time of symptom onset. In contrast, dogs with IVDE had significantly longer hospitalization periods and were more likely to be ambulatory at discharge. This could be since, in the case of IVDE patients, the postoperative period was monitored, and appropriate multimodal pain medication was administered until discharge, whereas in the ANNPE cases, an improvement in neurological status was usually observed, and pain therapy as well as monitoring is not necessarily indicated [19]. While the long-term outcomes did not significantly differ between the two groups, nor when the different Sharp and Wheeler grades was compared, ANNPE dogs were more prone to having persistent minor neurological deficits, which could impact the clinical prognosis. These results were consistent with previously published studies where 54–73% of ANNPE dogs had a successful outcome, which was defined as a partial or complete recovery of neurological function [20,22,26]. The peracute onset of clinical signs observed in ANNPE cases, as described in previous studies [3,19,22,26], was corroborated in this study.

This sudden onset was often associated with antecedent trauma or strenuous exercise. Patients enrolled in this study were plegic at the time of presentation, with no significant difference between the severity of the symptoms between the contusion and the compression groups (Table 2). In general, most of the dogs showed a lateralization of clinical signs, which was consistent with advanced diagnostic findings via CT or MRI. Although both groups were acutely paralyzed, dogs in the IVDE group generally showed a progression of neurological signs prior to presentation, whereas in patients with presumptive ANNPE, the clinical signs were stable or did improve 24 h after onset.

Notably, dogs with compressive IVDD often exhibited neurological deficits before paralysis, making it challenging to identify a specific trigger for the symptoms. These cases are initially presented to primary care veterinarians and receive conservative treatment protocols before referral. In contrast, the ANNPE cases exhibited a more peracute onset of paralysis, more rapid referral and an apparent history of external trauma frequently characterized by vocalization at onset [3,19].

Sex distribution did not significantly differ between the two groups, contradicting reports of a male predisposition in ANNPE patients [26,39,40], which could be due to the relatively small sample size in this study. In the study population, over 80% of the dogs in both groups displayed lesions in the T3-L3 spinal cord segment, aligning with previously published data [24]. Specifically, the most frequently affected intervertebral disc spaces observed in this study were T12/13, T13/L1 and L2/3, with no statistically significant difference between the two groups. While the exact cause of thoracolumbar intervertebral disc herniations remains elusive, it has been suggested that variations in biomechanical forces acting on the vertebral column, particularly at the interfaces between the relatively stable thoracic portion and the more dynamic cervical and thoracolumbar sections, may underlie the predilection sites for ANNPE [22,41,42].

All dogs in the study underwent a further diagnostic evaluation, including either a CT or MRI scan, depending on the suspected diagnosis. In cases with inconclusive outcomes in diagnostic imaging, a CT scan was followed by an MRI examination under the same anesthesia. CT scans offer advantages such as broader availability, lower costs, and shorter anesthesia durations. Additionally, most cases of compressive IVDD can be identified without the use of a contrast medium, making the CT scan a quick and sensitive screening tool to assess spinal cord compression. Given that spinal cord pathology is exclusively observable via MRI, it is the diagnostic imaging modality of choice for presumptively diagnosed ANNPE. It is important to note that a definitive diagnosis of ANNPE is only feasible through a pathological examination.

While the dogs with ANNPE generally displayed only mild spinal cord lesions on diagnostic imaging, those with IVDE were more likely to exhibit moderate changes. Interestingly, despite the imaging findings, the clinical signs of the dogs enrolled in this study were initially worse in the ANNPE group compared to the IVDE group, but the difference was not significant. This observation could be attributed to the distinct pathophysiology of the two conditions. In ANNPE, spinal cord compression is absent, and the clinical signs presumably result from secondary changes due to the high contusive energy involved in the incident [3]. In contrast, IVDE primarily leads to deficits due to progressive spinal cord contusion and compression [43].

Numerous studies suggest that a surgical approach via hemilaminectomy and mini-hemilaminectomy should be favored for non-ambulatory patients (paraparetic or worse) over conservative regimes in dogs with compressive IVDD [5,11]. Surgical intervention yields a diverse spectrum of outcomes for non-ambulatory patients, ranging from a 61% to 93% restoration of ambulation, in contrast to 10% to 62% in those treated conservatively [12,13,14]. The decision between a surgical or non-surgical approach is contingent upon multifaceted factors. Generally, ambulant dogs might be amenable to conservative management, but the risk of recurrence needs to be taken into consideration. Surgical intervention merits preference for patients with recurrent events, persistent or progressive neurological signs or when conservative management has failed to improve the condition [12,15,16,17,18]. It is important to note that, despite surgical intervention, only the compressive component of the condition can be addressed, as the contusive aspect and subsequent secondary changes cannot be directly treated.

Conversely, until now, current strategies for managing ANNPE predominantly center on conservative interventions, encompassing a 4–6-week regime of cage rest, short-term utilization of appropriate analgesics or anti-inflammatory drugs, a meticulous nursing protocol, and physiotherapy [19,20]. Depending on the patient’s neurological status, nursing care entails manual or instrumental bladder management if urinary dysfunction is suspected, repositioning non-ambulatory patients every 4 h to prevent decubital ulcers, in addition to nutritional and physical support [3,19]. Commencing physiotherapy early in the recovery phase is known to have a positive influence on the outcome and prognosis of regaining motor function [19,21]. The reported successful outcomes for ANNPE in dogs are highly variable, ranging from 66.7% to 100% [22,23,24,25]; however, a comparison of outcomes across studies is challenging due to the variations in the definition of a successful outcome, differing inclusion criteria, management protocols and the overall sample sizes in veterinary research.

All dogs diagnosed with IVDE underwent decompressive surgery, while those presumptively diagnosed with ANNPE received conservative treatment. Despite these divergent approaches, the outcomes in both groups exhibited no significant differences concerning independent ambulation at discharge, overall duration to achieve independent ambulation or long-term prognosis. It is worth noting that a well-known, short-term, life-threatening complication of spinal cord injuries is progressive myelomalacia, leading to ascending neurological signs with thoracic limb dysfunction, respiratory failure and death [44]. While myelomalacia is ultimately confirmed through histopathological examination, clinical signs and MRI findings can indicate presumptive progressive (ascending) myelomalacia [45]. In the present study, 6% of dogs with a Sharp and Wheeler grade of 5 exhibited severe myelomalacia symptoms (three in the contusion group and three in the compression group), which is a lower percentage than the previously reported range of 9–18% with the same severity of neurological deficits [46,47,48,49]. While several studies have addressed myelomalacia following severe spinal cord injury in dogs with acute intervertebral disc extrusion, limited knowledge exists regarding its development in ANNPE cases. Due to the small study population, a conclusive statement regarding myelomalacia in ANNPE cases cannot be made, necessitating further research.

In both groups, some dogs experienced persistent mild neurological deficits. According to the owners, these limitations did not affect the dogs’ quality of life (QOL). Dogs that exhibit persistent mild neurological deficits can still maintain a satisfactory QOL as a functional pet. These animals typically retain sufficient motor function and sensory perception to perform daily activities or engage in typical pet behaviors. However, it is important to acknowledge that such deficits may limit their ability to participate in high-performance activities or pursue a career in sports. The residual proprioceptive deficits, although mild, can impact the precision and coordination required for competitive sports, potentially predisposing the dog to further injury. Therefore, while these dogs can continue to live happy lives in a normal pet environment, their role in physically demanding activities should be carefully evaluated and potentially curtailed to ensure their long-term well-being. No significant statistical relationships were found between these neurological long-term effects and the initial clinical signs severity, the affected intervertebral disc level, or diagnostic imaging findings. At the time of the interview, eight dogs that were initially included in this study had been euthanized. Euthanasia in these cases was unrelated to the dogs’ initial neurological conditions and had no impact on the overall prognosis of the dogs in this study.

Several limitations should be acknowledged in this study. Firstly, the retrospective nature of this investigation hinders the implementation of a standardized case protocol. Retrieving essential data from the medical records was challenging, primarily owing to the involvement of multiple veterinarians in the patients’ treatment. In some cases, it was necessary to exclude specific parameters from the study protocol due to inconsistencies in the patients’ histories. Additionally, extra effort was required to objectify certain aspects of the documentation provided by individual veterinarians to ensure data reliability. Additionally, a systematic and objective neurological assessment approach was not available. Secondly, the study’s limited sample size, along with its single-center focus, restricts the generalizability of the findings to a broader population. Furthermore, it is unclear whether the random selection process of the contusion group may have contributed to variations in the results. Also, the definitive diagnosis of ANNPE is only possible postmortem, and a tentative diagnosis can only be made in the living animal based on clinical signs and diagnostic imaging, which makes a definitive classification challenging and misclassifying, in some cases, a possibility. Consequently, caution should be exercised in extrapolating these results to other settings. The authors acknowledge that the classification of the diseases in acompression and contusion group might not be so clearly evident. In all patients, the pathophysiology might involve compression, contusion and inflammation being present in different amounts in each individual patient. However, based on the description and the current knowledge about IVDD and ANNPE, it seems reasonable to assume that the compressive component in IVDD and the contusive component in ANNPE might be dominating the other pathophysiological pathways. Certainly, this issue should be taken into consideration when interpreting and validating the observed results.

Lastly, it is worth noting that some of the dogs that were euthanized for various reasons during the observation period may have potentially experienced recovery.

Despite these acknowledged limitations, the outcomes and prognosis for regaining walking ability in dogs with contusive IVDD did not differ significantly from those observed in dogs with compressive IVDD.

## 5. Conclusions

This study unveils the distinctions and parallels between dogs with acompressive or a contusive intervertebral disc disease. In accordance with previous assumptions, ANNPE-affected dogs were older, heavier and displayed unique clinical traits. Diagnostic imaging revealed pronounced changes in IVDE, contradicting clinical severity. Despite disparate treatments—surgical intervention for IVDE and conservative for ANNPE—the outcomes showed no significant differences. These insights contribute to the ongoing discourse on optimal treatment strategies for these paralytic conditions in dogs.

## Figures and Tables

**Table 1:** Classification of degree of neurological dysfunction based on Sharp and Wheeler [1].

Sharp and Wheeler	
**Grade**	**Description**
**1**	Pain only
**2**	Ambulatory paresis
**3**	Non-ambulatory paresis
**4**	Paralysis with deep pain sensation
**5**	Paralysis with loss of deep pain sensation

**Table 2:** Comparison of signalment and clinical variables between dogs with ANNPE (n = 45) and dogs with IVDE (n = 50).

	ANNPE	IVDE	*p*-Value
**Age (years)**	7 (0.2–13)	5.5 (3–15)	0.97
**Sex**			0.98
female	26 (58%)	29 (58%)	
male	19 (42%)	21 (42%)	
**Body weight (kg)**	23.6 ± 10.7	11.5 ± 7.6	<0.001
**Duration of clinical signs before examination (hours)**	3 (1–12)	16 (1–96)	
**Time frame from the onset of clinical signs to presentation**			<0.001
<12 h	45 (100%)	17 (34%)	
12–48 h	0 (0%)	29 (58%)	
>48 h	0 (0%)	4 (8%)	
**Event prior to the onset of clinical signs**			<0.001
Trauma	23 (50%)	2 (4%)	
Unknown	19 (42%)	43 (86%)	
Exercise	4 (8%)	5 (10%)	
**Vocalization at the onset of clinical signs**			<0.001
Yes	32 (71%)	4 (8%)	
No	13 (29%)	46 (92%)	
**Sharp and Wheeler grade at the time of examination**			0.16
Grade 4	33 (73%)	31 (62%)	
Grade 5	12 (27%)	19 (38%)	
**Deterioration of clinical signs before referral**			<0.001
No	39 (87%)	16 (32%)	
Yes	6 (13%)	34 (68%)	
**Medication received before referral**			
None	18 (40%)	15 (30%)	
NSAIDs	13 (28%)	24 (48%)	
Opioids	3 (7%)	5 (10%)	
Gabapentin/ Pregabalin	0 (0%)	5 (10%)	
Corticosteroids	12 (27%)	16 (32%)	
Unknown	8 (18%)	7 (14%)	
Others	11 (24%)	9 (18%)	
**Neuroanatomical localization of the lesion**			0.88
C1-C5	1 (2%)	1 (2%)	
C6-T2	0 (0%)	0 (0%)	
T3-L3	38 (84%)	44 (88%)	
L4-S3	6 (13%)	5 (10%)	
**Severity of the lesion**			<0.001
Mild	22 (49%)	6 (12%)	
Moderate	17 (38%)	33 (66%)	
Severe	6 (13%)	11 (22%)	
**Long-term outcome**			0.79
Successful	32 (71%)	40 (80%)	
Unsuccessful	3 (8%)	3 (7%)	
**Duration of hospitalization (days)**	2.5 (0–6)	3.7 (0–9)	<0.001
**Ambulatory at discharge**	5 (11%)	12 (24%)	0.10
**Time from onset to ambulation**			0.32
Within 14 days	14 (31%)	24 (48%)	
Within 2 months (first 14 days excluded)	12 (27%)	9 (18%)	
Within 3–8 months	6 (13%)	7 (14%)	
**Euthanized dogs**	10 (22%)	7 (14%)	0.3
Due to the owners’ request	6 (13%)	1 (2%)	
Failure of improvement after 14 days	1 (2%)	3 (6%)	
Suspected myelomalacia	3 (6%)	3 (6%)	
**Supportive treatment after diagnosis**			
Urinary catheter	20 (44%)	20 (40%)	
NSAIDs	39 (87%)	41 (82%)	
Opioids	25 (56%)	37 (74%)	
Gabapentin/Pregabalin	28 (62%)	39 (78%)	
Vitamin B	35 (78%)	29 (58%)	
Alpha1 Antagonists	25 (56%)	39 (78%)	
Parasympathomimetics	9 (20%)	27 (54%)	
Corticosteroids	2 (4%)	0 (0%)	
Physiotherapy	31 (69%)	28 (56%)	
Immobilization	21 (47%)	23 (46%)	
Others	8 (18%)	11 (22%)	

Legend: Acute non-compressive nucleus pulposus extrusion (ANNPE) n = 45; mineralized intervertebral disc extrusion (IVDE) n = 50; non-steroidal anti-inflammatory drugs (NSAIDs). Values represent the number of dogs (%), mean +/− SD or median (range) unless otherwise specified.

**Table 3:** Distribution of Sharp and Wheeler grades in relation to the neuroanatomical localization.

NA Lokalisation	ANNPE S/W 4	ANNPE S/W 5	IVDE S/W 4	IVDE S/W 5
**C1-C5**	1	-	1	-
**C6-T2**	-	-	-	-
**T3-L3**	29	9	28	16
**L4-S3**	3	3	2	3

Legend: NA = neuroanatomical localization; S/W = Sharp and Wheeler grade.

## Data Availability

The datasets used and/or analyzed during the current study are available from the corresponding author upon reasonable request.

## Abbreviations

The following abbreviations are used in this manuscript:

IVDE	Intervertebral disc extrusion
ANNPE	Acute non-compressive nucleus pulposus extrusion
CT	Computer tomography
MRI	Magnetic resonance imaging
